# OP51 Targeted Next-Generation Sequencing Gene Panel In Chronic Lymphocytic Leukemia Medical Care

**DOI:** 10.1017/S0266462325101098

**Published:** 2025-12-29

**Authors:** Audrey Nganbou, Denis-Jean David, Cédric Carbonneil

## Abstract

**Introduction:**

Chronic lymphocytic leukemia (CLL) is a malignant hematologic disorder that affects older adults. CLL is the most common leukemia in adults in the Western world. The genomic landscape of CLL is heterogeneous. The aim of this report was to identify the clinically relevant molecular alterations in CLL and define the role of targeted next-generation sequencing (NGS) in routine care.

**Methods:**

The evaluation included an analysis of systematic reviews, meta-analyses, clinical guidelines, and relevant molecular alterations using OncoKB™ and TOPOGRAPH classifications. Approvals from the French National Authority for Health Transparency Committee or compassionate use decisions from the French National Agency for Medicines and Health Products Safety were also considered.

**Results:**

Targeted NGS in CLL detected molecular alterations to determine prognosis and guide treatment decisions with a higher sensitivity than Sanger sequencing. The recommended analyses include:

- TP53 and IGHV mutations before first-line treatment;
- TP53 mutation before treatment modification in relapse cases;
- TP53, BTK, and PLCG2 mutations after treatment with BTK inhibitors;
- TP53 and BCL2 mutations after treatment with BCL2 inhibitors; or
- TP53, BTK, PLCG2, and BCL2 mutations after treatment with both BTK and BCL2 inhibitors.

**Conclusions:**

Targeted NGS is essential for managing CLL. The gene panel will be updated dynamically based on new scientific evidence and regulatory approvals.

